# Repetitive DNA in the pea (*Pisum sativum *L.) genome: comprehensive characterization using 454 sequencing and comparison to soybean and *Medicago truncatula*

**DOI:** 10.1186/1471-2164-8-427

**Published:** 2007-11-21

**Authors:** Jiří Macas, Pavel Neumann, Alice Navrátilová

**Affiliations:** 1 Biology Centre ASCR, Institute of Plant Molecular Biology, Branišovská 31, České Budějovice, CZ-37005, Czech Republic

## Abstract

**Background:**

Extraordinary size variation of higher plant nuclear genomes is in large part caused by differences in accumulation of repetitive DNA. This makes repetitive DNA of great interest for studying the molecular mechanisms shaping architecture and function of complex plant genomes. However, due to methodological constraints of conventional cloning and sequencing, a global description of repeat composition is available for only a very limited number of higher plants. In order to provide further data required for investigating evolutionary patterns of repeated DNA within and between species, we used a novel approach based on massive parallel sequencing which allowed a comprehensive repeat characterization in our model species, garden pea (*Pisum sativum*).

**Results:**

Analysis of 33.3 Mb sequence data resulted in quantification and partial sequence reconstruction of major repeat families occurring in the pea genome with at least thousands of copies. Our results showed that the pea genome is dominated by LTR-retrotransposons, estimated at 140,000 copies/1C. Ty3/gypsy elements are less diverse and accumulated to higher copy numbers than Ty1/copia. This is in part due to a large population of Ogre-like retrotransposons which alone make up over 20% of the genome. In addition to numerous types of mobile elements, we have discovered a set of novel satellite repeats and two additional variants of telomeric sequences. Comparative genome analysis revealed that there are only a few repeat sequences conserved between pea and soybean genomes. On the other hand, all major families of pea mobile elements are well represented in *M. truncatula*.

**Conclusion:**

We have demonstrated that even in a species with a relatively large genome like pea, where a single 454-sequencing run provided only 0.77% coverage, the generated sequences were sufficient to reconstruct and analyze major repeat families corresponding to a total of 35–48% of the genome. These data provide a starting point for further investigations of legume plant genomes based on their global comparative analysis and for the development of more sophisticated approaches for data mining.

## Background

Understanding evolutionary mechanisms shaping complex genomes of eukaryotes is impossible without thorough investigation of repeated genomic sequences [1-4]. This is especially obvious in higher plants, where repetitive sequences comprise up to 97% of nuclear DNA [5,6] and contribute significantly to the extraordinary genome size variation observed between different taxa [7-9]. However, the presence of numerous and sequentially diverse families of repetitive elements make their analysis a challenging task. Thus, the most widely used approaches to study the contribution of repetitive DNA to genome evolution are based on isolation and characterization of only a single or a small group of elements. These approaches have been valuable in following the fate of various repeats in a wide range of species [10-12]. However, they do not allow for the global comparative analysis of repeat profiles required for elucidating evolutionary trends on the whole genome level. The demand for a comprehensive repeat analysis prompted the development of a DNA microarray-based assay to study the occurrence of hundreds of repeats in twenty plant genomes [13]. Although successful, the microarray-based approach still suffered from several limitations including the need for a priori knowledge of the repeat sequences, the limited capacity of the array, and especially the inability to discover novel repeats for which there were no probes on the array.

The requirement for simultaneous determination of sequence composition and abundance of hundreds of repeat families is best fulfilled by analyzing the complete genome sequence; however, such data is available for only a limited number of model species. Alternatively, low-depth shotgun genomic sequencing can be used to survey the most abundant repeats, as was demonstrated for *Gossypium *species [9]. However, performing this type of survey using conventional approaches employing Sanger sequencing is still labor-intensive and requires considerable resources. The recent introduction of a massively-parallel pyrosequencing technology developed by 454 Life Sciences ("454-sequencing") has opened new possibilities for high-throughput genome analysis [14]. This approach allows parallel sequencing of hundreds of thousands of individual templates immobilized on microbeads, thus producing megabases of sequence data in a single run. It has been successfully applied to the sequencing of microbial genomes [15], the re-sequencing of mammalian genomes [16], and for transcript profiling [17]. Due to relatively short sequence read lengths (~100 nucleotides on average, or ~250 nucleotides with the improved version of the system), the technology is not yet suitable for de novo sequencing of complex genomes. However, it has a great potential for profiling repetitive sequences in these genomes, as the amount of produced sequence data is sufficient to get a representative overview of the most abundant genomic repeats. For example, a total of 30 Mb determined in a single sequencing run represents only 0.01-fold coverage of a hypothetical 3,000 Mb genome (this is about the haploid genome size of maize or cotton [18]), but provides 10-fold coverage of repeats occurring in the genome in 1,000 copies. The sum of 30 Mb is represented by a set of 300,000 sequence reads which are randomly generated from various genomic loci. Theoretically, they should contain fragments of a given repeat randomly sampled from its individual copies, and the frequency of these fragments in the sequence reads should be proportional to the genomic abundance of the repeat. Therefore, this amount of sequence data should be sufficient to reliably detect abundant (at least 500–1000 copies/1C) genomic repeats, and eventually reconstruct their consensus sequences by assembling the reads derived from their individual copies. Recently, this strategy has been successfully applied to repeat analysis in the 1,115 Mb genome of soybean [19].

Based on the theoretical considerations described above, we attempted to adapt parallel sequencing technology for the genome-wide characterization of repetitive elements in garden pea (*Pisum sativum *L.) and for comparative analysis of its repeat composition with other species. In addition to being a classical model for genetic studies, pea is also one of the model species used in our and other laboratories for studying the impact of repetitive DNA on legume plant genomes. Consequently, a set of well-characterized pea repetitive elements is available [20-24] which could serve as a control in evaluating the accuracy of the developed assays. Pea has a genome of 4,300 Mb/1C [18], which is about 10-fold larger than the genome size of rice or the model legume *Medicago truncatula*, and about 4-fold larger than the soybean genome. Compared to these species, it is rich in repetitive DNA, which was estimated to comprise 75–97% of its nuclear DNA [5,6].

Our initial experiments were aimed at evaluating the representation of known repeats in 454 sequence reads. Then we focused on the reconstruction of longer segments of repetitive sequences from multiple overlapping sequence reads, which provided a basis for their further characterization. These data were used to determine the genomic abundance and variability of the major repeat families present in the pea genome. Finally, we used the pea sequence data together with available soybean and *M. truncatula *sequences to perform comparative analysis of the repeat composition and abundance in these three legume species.

## Results

**Figure 1:**
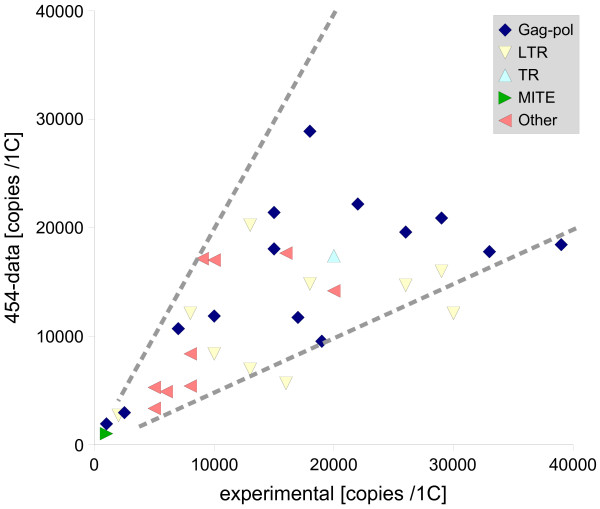
**Comparison of the repeat copy numbers determined experimentally to the estimates based on their frequency in 454 reads**. The comparison was performed for a set of previously characterized repetitive elements [20–24] including fragments of retrotransposon coding regions (gag-pol) and long terminal repeats (LTR), tandem repeats (TR), MITEs, and dispersed repeats of unknown origin (other). Detailed description of the repeats and their copy number estimates are available as Additional file 1. Dashed lines mark the area where the two estimates differ less than twofold.

A single 454 sequencing reaction with pea nuclear DNA resulted in 319,402 usable reads with an average length of 104 nucleotides, yielding a total of 33.3 Mb of sequence data. This is equivalent to 0.77%, or 1/129, of the haploid pea genome (1C = 4,300 Mb). Thus, in theory, repeats occurring at 1,000 copies or greater in the pea genome should be well represented in these sequences, as they should be covered on average by 7–8 sequence reads (1000/129 = 7.8) over their entire length. In order to test this assumption, we determined the representation of previously characterized pea repeats in the 454 data by sequence similarity searches against a database of individual sequence reads and calculated their average coverage by highly significant hits. As expected, low-copy or moderately repeated sequences, such as Zaba MITEs (50–500 copies/1C [22]), were represented by none or only a few hits. However, all 33 of the tested sequences with an abundance exceeding 1,000 copies/1C were well represented in 454 data, and their coverage by sequence reads was proportional to their abundance in the genome. The copy numbers of individual repeats calculated from the frequency of their occurrence in 454 reads were in a good agreement with estimates based on other experimental data (Fig. 1 and Additional file 1). These findings prove that the 454 data are representative for highly repeated sequences and thus can be further used for investigation and comprehensive description of this fraction of the pea genome.

### Reconstruction of genomic repeats by assembling 454 reads

The process of extracting repeat sequences from the set of 454 sequence reads was implemented using TGICL, a program package originally designed for clustering large EST datasets [25]. The procedure consisted of two steps: (i) clustering the reads based on their mutual similarities into groups of overlapping sequences, and (ii) assembling the reads within the clusters to get longer fragments (contigs) representing consensus sequences. Various clustering and assembly parameters were evaluated in order to get optimal performance with our dataset, which compared to ESTs differed in the short reading lengths and in their considerable sequence variability, reflecting the divergence between individual copies of repeated elements. While the clustering parameters allowed for grouping of relatively divergent sequences, the assembly process was more stringent, and thus typically generated multiple contigs from a single cluster.

The clustering resulted in 22,445 clusters, which were composed from two to thousands of reads. The assembly phase then yielded 25,384 contigs ranging in lengths up to 8,214 bp. Individual contigs were assembled from two to 4,327 reads, and the total number of sequence reads assembled into contigs was 233,303 (73 %). The contigs were characterized by their read depth (RD), expressing the average number of reads assembled over individual positions within the contig consensus sequence, and by their genome representation (GR), calculated as RD multiplied by the contig length. These values allowed us to rate the contig sequences based on their genomic copy numbers and proportion in the genome, respectively. Most contigs were short and composed of only a few reads, whereas 90% of assembled reads were assigned into a relatively small subset of 1,578 contigs with the highest GR, thus corresponding to highly repetitive sequences. Most repeats were represented as sets of overlapping contigs, the number of which was proportional to the sequence diversity of the repeat copies. Among the most important in terms of their genomic abundance were contigs that included coding sequences and conserved LTR regions of Ogre retrotransposons and other LTR-retroelements and of the satellite repeat PisTR-B (Additional file 2). Compared to the three previously sequenced pea Ogre elements [21], this set of Ogre-like contigs showed much higher sequence diversity, suggesting they represent several distinct Ogre subfamilies.

**Figure 2:**
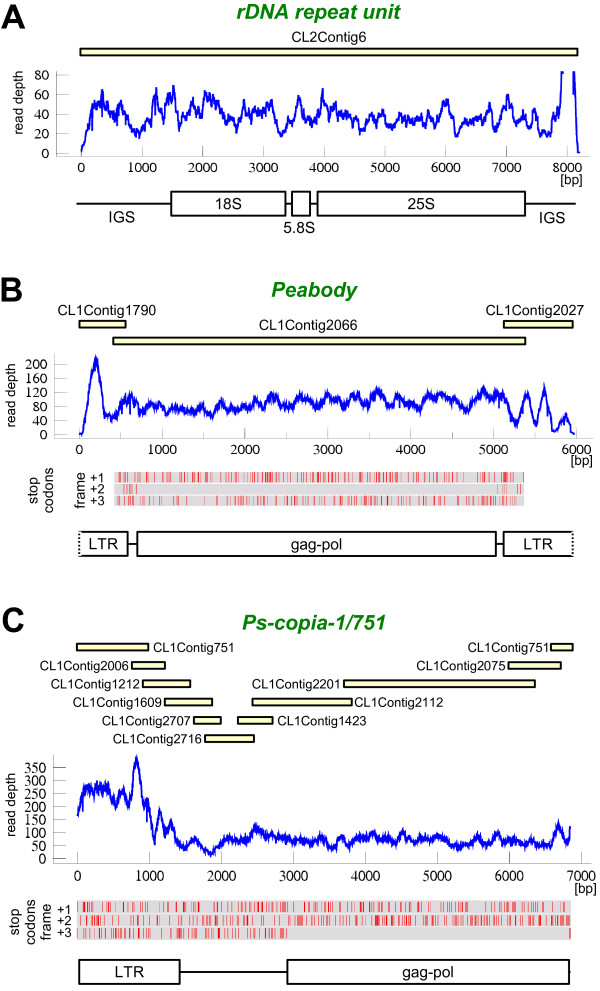
**Examples of repeat reconstruction from assembled 454 reads**. (**A**) The complete rDNA coding region including genes for 18S, 5.8S and 25S rRNA and parts of the large intergenic spacer (IGS) was reconstructed as a single contig (CL2Contig6). The graph shows the read depth (number of assembled reads) along the contig sequence. (**B**) Reconstruction of the Ty3/gypsy retroelement Peabody, including most of its long terminal repeat (LTR) sequence and complete polyprotein-coding region (gag-pol). (**C**) A novel Ty1/copia element Ps-copia-1/751, reconstructed from ten overlapping contigs. The region devoid of stop codons encoding gag-pol was identified in frame +3. Yellow bars depict length and relative positions of the overlapping contigs, stop codons are represented by red vertical lines.

The longest contig (CL2Contig6) represented a 8,214 bp fragment of the rDNA gene cluster, including the complete 18-5.8-25S gene sequences (5,820 bp) surrounded by 3' and 5' parts of large intergenic spacer (Fig. 2A). Comparison to previously published partial pea rDNA sequences revealed its identity with a 1,723 bp fragment of the 18S rRNA gene [GenBank: U43011] and 99.5% similarity to a 620 bp sequence region including the 5.8S rRNA gene and both internal transcribed spacers [GenBank: AY839340].

The contig with the highest genome representation was a 5,097 bp fragment of the LTR-retrotransposon Peabody [GenBank: AF083074]. This contig (CL1Contig2066) had a read depth corresponding to 11,000 copies/1C and was estimated to make up 1.3% of the pea genome. It included a complete internal retrotransposon region surrounded by parts of LTR sequences, which could be further extended by finding and aligning overlapping contigs (Fig. 2B). The internal region contained a gag-pol coding sequence (4,368 bp) devoid of stop codons.

The highest read depth (193 reads, corresponding to about 25,000 copies/1C) was found for a 1,213 bp contig CL1Contig751 representing the LTR sequence of a novel Ty1/copia element designated Ps-copia-1/751. The element reconstruction from ten overlapping contigs resulted in identification of the complete LTR and most of the internal region including open reading frame encoding gag-pol polyprotein (Fig. 2C).

In addition to highly repeated sequences it was also possible to at least partially reconstruct less abundant repeats, many of which were novel to the pea genome. For example, an over 7 kb region of a MuDR-like DNA transposon, including 2 putative coding regions, could be reconstructed from 25 overlapping contigs. MuDR elements were estimated to occur in about 2,200 copies in the pea genome, and similar abundance was also found for another DNA transposon family, En/Spm, for which it was possible to reconstruct a 3 kb fragment of the transposase-coding region (not shown).

**Figure 3:**
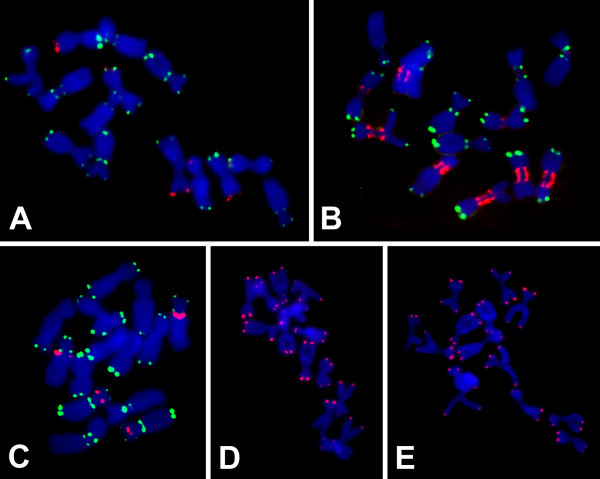
**Localization of newly identified tandem repeats on pea metaphase chromosomes using FISH**. Red signals show chromosomal localization of the tandem repeats TR-9 (**A**), TR-11 (**B**), TR-3 (**C**), and telomere-like sequences (TTTAGG)_n _(**D**), and (TTAGG)_n _(**E**). Preparations on panels **A-C **were simultaneously hybridized to PisTR-B probe (green) to discriminate individual chromosome types [20]. Chromosomes were counterstained with DAPI (blue).

The clustering and contig building procedure was also found useful for identifying novel tandemly repeated sequences. Assembling overlapping reads into longer contigs facilitated reconstruction of repeats with monomers exceeding the length of single reads and allowed their identification based on the tandem subrepeats present within the contigs. A number of contigs representing potential satellite repeats with monomers from 50 to 867 bp were identified; except for the previously described PisTR-B satellite [20], they all represented novel sequences. Fourteen of the most abundant repeats (Table 1) were used as probes for in situ hybridization on pea mitotic chromosomes in order to test if they have a genomic distribution typical for satellite DNA. Such hybridization patterns, consisting of signals concentrated into limited number of spots corresponding to long arrays of the satellite sequences, were observed for thirteen repeats, whereas only one produced dispersed signals (Fig. 3A–C and Table 1). The signals occurred mostly in (peri-) centromeric and terminal chromosome regions, and each repeat displayed a specific hybridization pattern. No typical centromeric satellite repeats were found, although the repeat TR-11 strongly labeled central centromeric regions in five out of the seven chromosome pairs (Fig. 3B).

**Table 1:** Newly identified tandem repeats with high abundance in the pea genome

Repeat	Monomer [bp]	Cluster	Abundance^(a)^		Localization on chromosomes^(b)^							Note
					
			[%]	CN	1	2	3	4	5	6	7	
TR-1	867	CL14	0.14	7,000	PC	-	C, T	PC,C	-	-	-	
TR-2	~440	SCL5	0.29	28,000	-	PC	-	-	C	PC	-	
TR-3	82	CL19	0.10	51,000	-	-	PC	I	-	-	-	Fig. 3C
TR-4	172	CL16	0.09	22,000	-	-	-	-	-	PC	-	
TR-5	54	CL52	0.04	35,000	-	PC	-	-	-	-	-	
TR-6	245	CL65	0.04	7,000	-	-	-	-	PC,C	-	-	
TR-7	164	CL49	0.05	13,000	PC,C	-	-	-	-	-	-	
TR-8	342	SCL58	0.04	5,000	~	~	~	~	~	~	~	Dispersed
TR-9	189	CL68	0.04	8,000	T	T	-	-	-	-	T	Fig. 3A
TR-10	659	CL78	0.03	2,000	-	-	-	-	PC,C	-	-	
TR-11	~510	CL9	0.20	17,000	C	C	-	-	C	C	C	Fig. 3B
TR-12	~120	CL92	0.02	7,000	I	PC, I	I	C, I	I	I	I	
TR-14	193	CL124	0.02	5,000	-	-	-	T	-	-	-	
TR-17	191	CL193	0.01	3,000	-	-	-	-	-	-	I	

^(a) ^Repeat abundance is expressed as its proportion in the genome (in percents of the genome size) and as copy number of monomers per haploid genome (CN).

^(b) ^Positions of FISH signals on individual chromosomes (n = 7). C, centromeric; PC, pericentromeric; I, intercalary; T, (sub-)telomeric.

### Composition of the most repetitive fraction of the pea genome

Using a combination of various tools including sequence similarity searches, conserved protein domain detection, and structure analysis of the contigs, it was possible to assign the most abundant reconstructed sequences into specific classes of repetitive elements (Table 2 and Additional file 3). It was found that the majority of pea repetitive DNA is made up of LTR-retrotransposons, with the most prominent group being the Ty3/gypsy-like Ogre elements, which alone were estimated to constitute 20–33% of the genome. Investigation of the sequence variability of contigs representing overlapping fragments of Ogre sequences revealed the presence of several subfamilies of these elements. Further analysis based on quantification of the sequence reads from contig regions that include the primer binding site (PBS) typical for Ogres [26] and comparison of their surrounding sequences confirmed the occurrence of three distinct subfamilies. It also provided an estimate of their abundance, which was about 30,000 elements for each of the two major subfamilies and 8,000 copies/1C for a minor one.

**Table 2:** Repeat composition of the pea genome estimated from genomic abundance of reconstructed contigs

	Genome representation^a^			Copy numbers^b^		
		
	Clusters (contigs)	Total GR	Genome proportion [%]	Copies/1C (domain)	Element length [bp]	Genome proportion [%]
Retroelements						
Ty3/gypsy						
Ogre-like	77 (632)	6,754,965	20.30	65,000 (RT, PBS)	22,000	33.26
						
Peabody	5 (42)	703,484	2.11	16,000 (RT)	8,000	2.98
PIGY	23 (70)	361,389	1.09	4,700 (RT)	13,600	1.49
Cyclops	15 (52)	221,925	0.67	2,700 (RT)	12,300	0.77
Ty1/copia						
Ps-copia-1/751	6 (14)	690,339	2.07			
full-length				8,000 (RT)	8,000	1.49
solo-LTR				9,000	1,400	0.29
SIRE	9 (87)	548,280	1.65			*(1.65)*
PDR	6 (24)	214,755	0.65	1,100 (RT)	4,000	0.10
Other copia	16 (26)	101,805	0.31			*(0.31)*
Other RE	17 (111)	878,773	2.64			*(2.64)*
						
DNA transposons						
MuDR	7 (24)	68,247	0.21			*(0.21)*
En/Spm	5 (9)	50,845	0.15			*(0.15)*
						
Tandem repeats						
45S rDNA	1 (6)	348,987	1.05	5,300	8,680	1.07
5S rDNA	1 (3)	13,134	0.04			*(0.04)*
PisTR-B	1 (7)	147,113	0.44			*(0.44)*
Other satellites	16 (73)	381,148	1.15			*(1.15)*
						
		**Total :**	**34.5 %**			**48.0 %**

^a^Genome proportion of individual groups of repeats was estimated from the sum of genome representation (GR) values of corresponding clusters of contigs.

^b ^Estimates based on genomic copy numbers inferred from the read depth of contigs including conserved sequence domains (RT, reverse transcriptase; PBS, primer binding site). Genome proportion of the repeats was calculated using the length of the complete elements and therefore it is provided only for repeat families with known full-length copies in the pea genome.

Although other retroelement families were found in considerably smaller numbers, there were several elements which made up significant proportions of the genome. They included Peabody, which made up 2–3% of the genome and displayed very low sequence variability suggesting its recent amplification. Other important groups of Ty3/gypsy elements were represented by PIGY [24] and Cyclops [27]. Ty1/copia retrotransposons were found to be less frequent, being represented by PDR [28] and a group of SIRE1-like sequences [29]. However, the most abundant was a novel element, Ps-copia-1/751 (Fig. 2C), which made up about 2% of the pea genome. The LTR sequence of this element was estimated to occur in at least 25,000 copies in the pea genome, whereas other regions are less frequent (about 8,000 copies/1C), thus indicating the existence of a large number of solo-LTRs derived from this element (Table 2).

**Figure 4:**
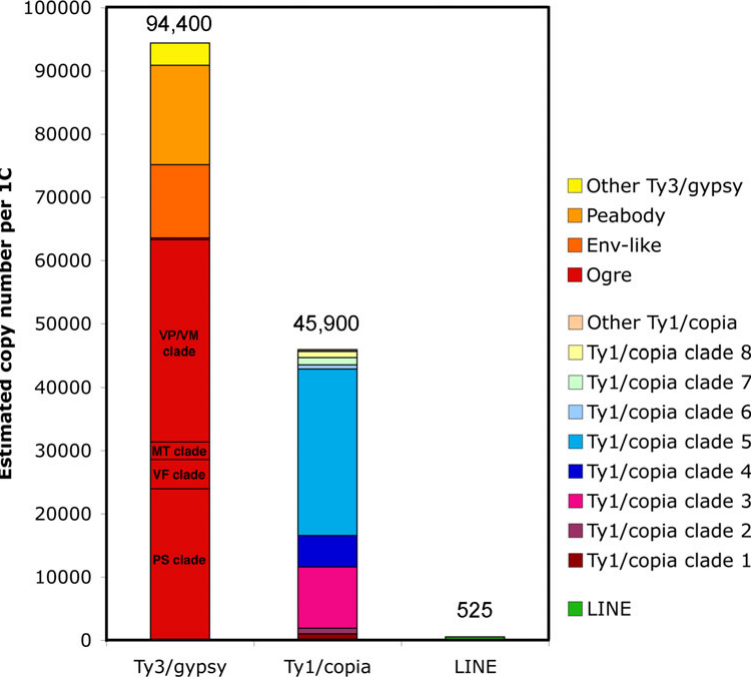
**Classification and copy numbers of pea retrotransposons based on their reverse transcriptase domains**. Individual groups of retrotransposons were classified according to their position within the phylogenetic trees or based on their best BLAST hits (Additional files 4 and 5). Ogre retrotransposons were further classified into several subfamilies as shown in the Additional file 4.

Overall diversity of retrotransposons was also studied by their classification based on the RT domains, which are conserved enough to be reliably identified based on protein similarity searches with RT domains from already known retrotransposons. The searches resulted in finding 452 contigs with similarity to RT domains (E-value <= 0.001). Of them, 222 contigs were related to Ty1/copia, 217 to Ty3/gypsy, and only 13 to LINE retrotransposons. Only four Ty1/copia-like and seven Ty3/gypsy-like contigs spanned full-length RT domains as defined by [30] while the rest contained partial sequences due to the short length and/or termination within the RT domain. Phylogenetic analysis of RT sequences belonging to Ty1/copia and Ty3/gypsy groups revealed that most Ty3/gypsy-like RT domains are related to the previously described pea retrotransposon families Ogre, Peabody, PIGY and Cyclops (Additional file 4). On the other hand, Ty1/copia-like RT domains were related to a greater number of retrotransposon families, most of which have not been previously identified in the pea genome (Additional file 5). RT domains were also used to estimate the copy number of retrotransposons present in pea. These elements were estimated to occur in about 141,000 copies/1C, of which 46,000 (32.6 %) belong to Ty1/copia, 94,000 (67%) to Ty3/gypsy and ~500 (0,4%) to the LINE group (Fig. 4). The highest copy number was estimated for Ogre retrotransposons, which amplified themselves to about 64,000 copies. More detailed analysis of Ogre-like RT domains confirmed the occurrence of distinct Ogre subfamilies sharing their best similarities with elements from different branches of the Ogre clade (Additional file 4). The most abundant were subfamilies from PS and VP/VM branches having about 24,000 and 32,000 copies, respectively (Additional file 4 and Fig. 4). Thus, the total copy number of Ogre elements as well as the abundance of the two major subfamilies were in agreement with estimates based on the abundance of PBS sites described above. Two other clades of Ty3/gypsy retrotransposons, Peabody and envelope-like retrotransposons (including PIGY and Cyclops), were estimated to have 16,000 and 12,000 copies, respectively. Although Ty1/copia retrotransposons were less frequent than Ty3/gypsy, elements from clades 3 and 5 (Additional file 5) reached high copy numbers (10,000 and 26,000 copies, respectively; Fig. 4). Interestingly, clade 3 contained elements from Graminae species such as barley (BARE-1), rice (RIRE-1), and oats (OARE-1) clustered together with a newly identified pea element Ps-copia-1/751. Clade 5 contains elements similar to SIRE1, an envelope-like retrotransposon from soybean [29]. It is not clear yet, however, whether all elements within this clade are genuine envelope-like retrotransposons, i.e. whether they all bear envelope-like gene.

Compared to the sum of retroelement sequences, other classes of repeats represented significantly smaller parts of the pea genome (Table 2). Identified DNA transposons did not exceed 0.5%, and tandem repeats including rDNA gene clusters and various satellite sequences represented about 2.5% of the pea genome. We have also investigated the abundance of micro- and minisatellite repeats with monomers from 2 to 10 bp. Since these repeats usually occur in the genome as short stretches of repeated monomers dispersed within unrelated sequences, we analyzed their frequency in unassembled sequence reads instead of contigs. When considering arrays of at least five consecutive monomers, microsatellites (AAT)_n_, (AT)_n_, and (AG)_n _were found to be most frequent, occurring in about 80,000 genomic loci. Other microsatellite motifs were less abundant and there were differences over two orders of magnitude in the frequency of various microsatellites within the reads (data not shown).

Special attention was paid to detection of telomeric repeats. The Arabidopsis-type telomeric sequence (TTTAGGG)_n _[31] was present in 14 reads, with an average number of 18 repetitions per read. This gives a rough estimate of about 229 kb of this sequence in the pea genome, and agrees with experimental observations of this repeat at termini of all chromosomes (data not shown). However, two additional telomere-like repeats were found in the analyzed reads – (TTAGG)_n _and (TTTAGG)_n_. Although both were less frequent than the Arabidopsis-type repeat, their sequences also spanned the whole reads, suggesting they occur in the genome as longer contiguous arrays. FISH experiments using labeled oligonucleotide probes confirmed this presumption, revealing the presence of both sequences at the termini of all pea chromosomes (Fig. 3D–E). To check for specificity of this assay, control experiments were run using probe sequences differing in a single-base substitution within the repeat monomer ((TTACG)_n _and (TTTACG)_n_). These sequences were not present in the 454 reads and corresponding probes gave no hybridization signals on mitotic chromosomes (data not shown).

### Comparative analysis of the repeat composition of pea, soybean and *M. truncatula *genomes

**Figure 5:**
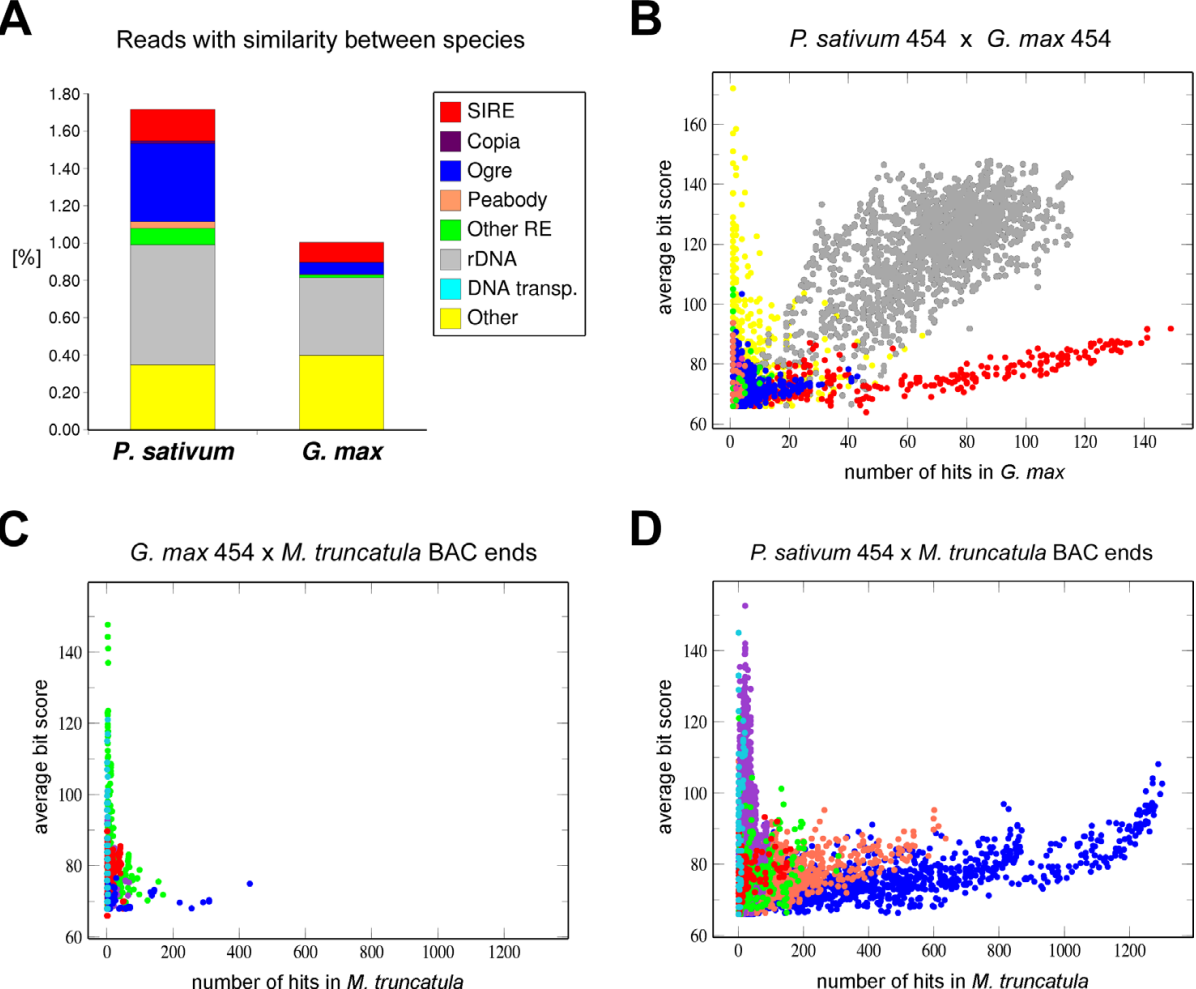
**Repeat families common to *P. sativum*, *G. max *and *M. truncatula *genomes**. **A. **Proportion of sequences shared between pea (*P. sativum*) and soybean (*G. max*). The graph shows percentage of 454 reads with significant similarity to sequences from the other species. The contribution of various repeat families is displayed in different colors as indicated on the legend. **B-D**. Repeat families conserved between pea and soybean (**B**), soybean and *M. truncatula *(**C**), and pea and *M. truncatula *(**D**). Dots on the scatter plots represent 454 reads from one species with similarity to sequences from the other species. Positions of the dots along the axes correspond to the number of significant hits obtained for each 454 read and to their average similarity expressed as BLAST bit score. Repeat families are displayed in the same colors as on panel A; rDNA and unidentified sequences were omitted from panels C and D.

In addition to the identification of the major components of pea repetitive DNA, we were interested in using the 454 sequence data for comparing the pea genome composition to that of related legume species. We primarily focused on soybean (*Glycine max*), as it was the only species for which whole genome shotgun reads produced by the same technology were available. The soybean 454 data included 718,589 reads with an average length of 109 nucleotides [19]. To investigate which sequences are shared between these two genomes we identified and analyzed pea 454 reads producing significant similarity hits (E-value <= 1e^-10^) in BLAST searches against a database of the soybean 454 reads. A total of 5,482 pea reads (1.7%) matched soybean sequences; in the soybean dataset, 7,209 reads (1.0%) gave significant hits with pea. The composition of these sequences is summarized in Fig. 5A–B. The largest fraction of the matching reads belonged to rDNA sequences, which also displayed the highest sequence similarity between the species (reflected by high BLAST bit scores on Fig. 5B). Ogre elements, representing the most abundant repeats in the pea genome, were also identified in soybean but in much smaller numbers. On the other hand, the two species were found to share relatively large population of SIRE1 sequences. Interestingly, the other abundant families of pea Ty1/copia elements were absent or gave only a few hits in the soybean genome.

In order to compare genome composition of pea and soybean with the legume model species *Medicago truncatula*, we employed the same strategy as above, except for using a set of 197,570 *M. truncatula *BAC-end sequences instead of 454 data. We found that pea genome contains considerably more repeats similar to *M. truncatula *sequences than soybean. A total of 15,510 (4.9%) pea reads gave significant matches with *M. truncatula *and included all major pea repeat families (Fig. 5D). However, there were differences in genomic proportion and sequence conservation of individual repeats between pea and *M. truncatula *genomes. For example, abundant pea Ogre sequences were also frequently found in *M. truncatula *but their sequence similarity was relatively low, whereas Ty1/copia elements (represented mostly by Ps-copia-1/751 family) produced much fewer hits but displayed higher average sequence similarity (Fig. 5D). Contrary to pea, soybean repeats were only poorly represented in *M. truncatula*. About 2.1% of soybean 454 reads matched *M. truncatula *sequences, however, except for rDNA (0.25%) no major repeat family was found to be shared by these two species (Fig. 5C). In summary, these results indicate similarity in repeat composition between pea and *M. truncatula *genomes, and considerable sequence divergence of most repeat families between these two species and soybean.

## Discussion

The rationale behind adapting 454 sequencing to repeat profiling in complex plant genomes is that it provides efficient sequence sampling from a high number of independent genomic loci. The amount of generated sequence data is large enough to include multiple reads from highly repeated elements, thus allowing evaluation of their abundance and sequence composition. However, required prerequisites for the use of 454 sequencing for repeat quantification and reconstruction are that the template sampling is random and that the sequencing does not introduce a bias towards certain sequences. In this study, we addressed these questions by comparing repeat copy number estimates obtained using experimental approaches [20-24] with the estimates based on the frequencies at which these repeats occur in 454 reads. The values were within a two-fold difference range for most of the repeats, and they never differed more than 2.8-fold. The observed discrepancies can be explained by principal limitations of both analytical approaches. The experimental quantification was based on DNA-DNA hybridizations, which may bias estimates if the probe fragment spans sequence regions differing in genomic abundance. In that case, hybridization signal is primarily determined by only a part of the sequence, but it is considered to be representative for the whole probe in subsequent calculations. This is not the case for the calculations of genome representation based on sequence coverage by 454 reads, which are performed separately for each nucleotide of the sequence in question. On the other hand, sensitivity and specificity of sequence similarity searches employed for this analysis can be partially affected by the algorithm and the similarity threshold values used for the assay. Thus, taking into account these limitations, we consider the experimental and 454 data to be in a good agreement.

Reconstruction of the repetitive element sequences from 454 reads represents a difficult task, complicated by the short length of the reads and considerable sequence diversity of individual genomic copies of the repeat. A similar problem has been successfully addressed by Li and co-workers [32], who aimed at the recovery of ancestral sequences for rice mobile elements from whole genome shotgun sequences. They developed an algorithm based on short oligomer (K-mer) frequency analysis for repeat identification and reconstruction. However, the program implementing this analysis was designed for processing conventional sequence reads of at least several hundred nucleotides in length and could not be adapted to work with the short 454 sequences. Thus, we used a different strategy, employing sequence-similarity based clustering of the reads followed by assembling them into contigs representing reconstructed fragments of the genomic repeats. Although the TGICL program package used to perform this analysis was originally designed for clustering ESTs [25], it provides a number of customizable parameters, which after proper adjustment resulted in the desired performance with our data. It should be noted, however, that even with these settings, most repeats could not be reconstructed as a single contig spanning their full-length sequences and including most of the sequence reads. This is mainly due to the occurrence of multiple subfamilies of the repeats in the genome and the presence of poorly conserved repeat regions. On the other hand, highly conserved rDNA repeats could be reconstructed as a single contig and their consensus was in excellent agreement with the sequences obtained by conventional cloning and sequencing of pea rDNA fragments.

Instead of performing direct contig assembly from all 454 reads [19], we preceded the assembly with a clustering step, which resulted in partitioning the read collection into groups of overlapping sequences. In addition to reducing the computational complexity of the assembly step, this approach also allowed the classification of contigs based on their cluster of origin. In principle, multiple contigs resulting from the assembly of reads from the same cluster should represent overlapping fragments and sequence variants of the same repeat family. Whereas this was true for many repeat families, there were also clusters including reads from several unrelated repeats. This is likely due to the existence of reads containing parts of two different repeats which act as a bridge to join groups of unrelated sequences during the transitive closure clustering procedure. Such reads can, for example, originate from insertion sites of mobile elements, which are numerous in the genome and often located within other repetitive sequences. This assumption is supported by our results, where this problem occurred in the largest cluster (CL1), which originated from at least five different families of retroelements. Although such clusters can be subsequently broken into smaller sets of overlapping contigs (see Methods), we plan to avoid this problem in the future by developing algorithms for identification of such "hybrid" reads and their elimination from the clustering procedure.

Our results have shown that low-depth genome sequencing using massively parallel technology provides sufficient sequence data for comprehensive repeat characterization even in a relatively large plant genome. Compared to the only other study on this topic, employing 454 sequencing for repeat analysis in soybean [19], the pea 454 sequences used here provided considerably smaller genome coverage (0.77% vs. 7% in soybean) due to the 4-fold difference in genome size between these species and the smaller reaction scale used in the pea sequencing. Still, it was possible to characterize repeats constituting 35–48% of the pea genome and including all major classes of repetitive DNA. On the other hand, considering the estimated 75–97% proportion of repeats in the genome [5,6], relatively large fraction of the repeats remained uncharacterized. Reassociation kinetics studies of pea genomic DNA [6] as well as observations from other species [33] indicate that this fraction includes diverged, low-copy remnants of ancient repeats ("fossil-repeats"). Such repeats are below the sensitivity limit of our analysis due to their high sequence variability and low copy numbers.

Similar to most higher plants studied so far [34-36], LTR-retrotransposons were found to be the major component of pea repetitive DNA. Ty3/gypsy elements were present in twice as many copies as Ty1/copia and constituted an even larger portion of the genome (24–39%, vs. 5% spanned by Ty1/copia) owing to much longer element sequences. The prevalence of Ty3/gypsy elements over other groups of retroelements was observed in other plant genomes including rice [37] and *Vicia *sp. [38], and their differential proliferation substantially contributed to the genome size variation among related species [9]. In pea, most of the Ty3/gypsy sequences were classified as Ogre-like retrotransposons, a distinct evolutionary lineage of giant elements occurring in a range of dicot plants including the genera of Leguminosae, Solanaceae, and Salicaceae [26]. Ogre elements were found to play an important role in genome evolution of *Vicia*, a genus closely related to *Pisum*. They were differentially amplified in individual species, with the highest abundance in *V. pannonica *where their recent expansion to 10^5 ^copies/1C increased the genome size by more than 50% [8]. Contrary to *V. pannonica*, the Ogre population in pea is not as homogeneous but it occurs as several distinct subfamilies differing in their sequences. This suggests that the evolutionary history of Ogre elements in pea was more complex and included processes of amplification and diversification of the elements. Although they are the most abundant, Ogre elements are probably not the only Ty3/gypsy elements with a significant impact on pea genome evolution. For example, Peabody elements were found to be very conserved in their nucleotide sequences, implying their recent amplification.

Compared to Ty3/gypsy elements, Ty1/copia represented a much smaller portion of the genome but occurred in a larger number of different families. Intraspecific heterogeneity of the Ty1/copia population, resulting from the presence of divergent families, was reported in a number of other species [39]. Interestingly, these families are well conserved across different taxa in spite of their ancient origin before the divergence of monocots and dicots [40]. This is also true for the pea Ty1/copia sequences, which in some cases, show high similarity to elements from phylogenetically distant species (Additional file 5). A typical example is the most abundant family Ps-copia-1/751 with strong similarity to monocot elements RIRE-1 and BARE; moreover, the high proportion of solo-LTRs derived from Ps-copia-1/751 suggests its long presence in the pea genome.

Whereas the general composition of dispersed repeats represented by various groups of mobile elements resembled that of other plants with complex genomes, our analysis revealed surprising diversity of tandem repeats in the pea genome. In addition to the previously described PisTR-A and PisTR-B repeats [20], thirteen novel families of abundant tandem repeats showing genomic organization typical for satellite DNA have been identified. This contrasts with most plants studied so far for which only a single or a few satellites are known [41]. However, whether this is a specific feature of the pea genome or simply a consequence of highly efficient tandem repeat identification employing 454 data remains to be seen after more species will be analyzed using this technology. Nevertheless, the availability of such a rich set of satellite repeats differing in monomer length, sequence, and chromosomal localization makes pea an attractive model for studying this type of repeated DNA. For example, our previous investigation of PisTR-B repeats using COD-FISH revealed uniform orientation of its monomers with respect to telomeres on most subtelomeric loci [42]. Extending this study to other satellite families should show if this is a general feature of the satellite arrangement at pea chromosome termini. Moreover, the wealth of sequence data obtained in this study will allow detailed characterization of sequence variability of individual families and testing if it correlates with the repeat chromosomal localization as was shown for other species [43]. Yet another interesting question concerns the possible lack of a satellite repeat conserved among pea centromeres. Although all pea centromeres seem to contain satellite DNA (Table 2), no family of the newly identified tandem repeats occupies all centromeres as is common in most plant species characterized so far [44,45]. This might either suggest that the genuine centromeric satellite has not been identified in our sequences or that the centromeric sequences in pea underwent less extensive homogenization among non-homologous chromosomes.

In addition to the Arabidopsis-type telomeric repeats, two other variants of telomeric minisatellite sequences were identified in the pea genome. Although they were both localized at chromosome termini along with the Arabidopsis-type sequences, their origin and role in telomere maintenance are unclear. Occurrence of the mixed minisatellite telomeric motif was reported from several plants and could be attributed to low fidelity of telomerase [46]. The relatively small number of 454 reads containing telomeric repeats did not allow us to perform a thorough investigation of their variability; however, there were several reads including non-perfect or mixed repeat motifs which could support this hypothesis (data not shown). On the other hand, both alternative repeats were also found to form arrays spanning the whole read lengths, which indicates their at least partial arrangement in longer homogeneous arrays.

## Conclusion

This work provided the first detailed survey of repetitive sequences in garden pea. It confirmed the expected high proportion of repeats in the pea genome and revealed that it is mostly attributed to various families of mobile elements. Amplification of a few groups of Ty3/gypsy elements, especially those belonging to Ogre-like retrotransposons, contributed the most to the bulk of pea repeats. Ty1/copia elements were found to be less abundant but more diverse in their sequences, occurring in a number of distinct (sub-)families. Other mobile elements including non-LTR retrotransposons (LINEs) and DNA transposons of the MuDR and En/Spm families were also detected. However, their total abundance did not exceed thousands of copies per haploid genome, thus representing only a minor part of pea nuclear DNA. Tandem repeats identified in the pea genome included microsatellites, three variants of telomeric minisatellites, and exceptional diversity of satellite repeats. Localization of newly identified satellite sequences on mitotic chromosomes revealed their family-specific hybridization patterns, providing novel cytogenetic landmarks for chromosome mapping.

Although the presented analysis yielded a wealth of information about the repeat composition of the pea genome, it was also useful in uncovering various limitations of our analytical approaches, which should be improved in the future. In addition to these improvements, a number of novel ways to utilize 454 data in plant genome analysis can be envisioned. They include, for example, repeat masking in genome sequencing projects, detailed investigation of intra- and intergenomic repeat variability, and identification of conserved non-coding regulatory sequences. Of special interest is the application of this technology to comparative genomics in a wide range of species, which should provide key information for understanding evolutionary patterns of repetitive sequences and their impact on genome evolution. Our results demonstrated the feasibility of this approach and revealed that in spite of differences in abundance of individual families, the repeat composition in pea and *M. truncatula *is similar, whereas both these species share only a few conserved repeats with soybean.

## Methods

### Genomic DNA isolation and 454 sequencing

Seeds of garden pea (*Pisum sativum *L. cv. Carrera) were obtained from the Plant Breeding Station at Boršov, Czech Republic. The DNA was extracted from purified nuclei in order to minimize contamination with chloroplast and mitochondrial genomes. The nuclei were isolated from young leaves by grinding 5 g of the tissue in liquid nitrogen, followed by 5 min incubation in 35 ml of ice-cold H buffer [47]. The homogenate was filtered through 48 μm nylon mesh, adjusted to 35 ml volume with 1 × H buffer, and centrifuged at 200 × g for 15 min at 4°C. Pelleted nuclei were resuspended and centrifuged using the same conditions once in 35 ml of H buffer, and once in 15 ml of TC buffer (50 mM TRIS-HCl pH 7.5, 75 mM NaCl, 6 mM MgCl_2_, 0.1 mM CaCl_2_). The final centrifugation was performed for 5 min only and the nuclei were resuspended in 2 ml of TC. DNA was released by incubating the nuclei with 40 mM EDTA, 0.2% SDS and 0.25 μg/μl of proteinase K for 4 hours at 37°C and purified by phenol extraction and ethanol precipitation. The sequencing was performed by 454 Life Sciences (Branford, CT, USA) using the GS-20 instrument and yielded 322,396 quality filtered sequence reads with the average length of 104 bp. The reads were deposited into NCBI Short Read Archive [48].

### Processing and initial analysis of 454 reads

The reads were screened for their similarity to adaptors and primers used in 454 sequencing using BLAST [49] and 595 reads producing blastn hits with expectation (E) value < 1e^-15 ^were removed from the set. The same procedure was repeated to detect contamination with organellar sequences, removing 2,322 reads with similarity to plant chloroplast genomes and 77 reads similar to mitochondrial DNA. The remaining 319,402 reads were used for all subsequent analyses. These reads included a total of 33.3 Mb, corresponding to 1/129 of the pea haploid genome size (1C = 4,300 Mb [18]). Thus, the calculated repeat copy numbers were multiplied by 129 in order to compensate for the actual genome coverage.

To determine the representation of previously characterized pea repeats in 454 sequences, these repeats were used as queries in blastn searches against a database of the 454 reads, using E-value cutoff of 1e^-10^. BLAST outputs were parsed using a BioPerl [50] script determining the number of similarity hits at all positions along the sequences, calculating their average numbers and the corresponding estimates of genomic copy numbers. The filtered set of 454 reads used for this analysis is available for BLAST searches at our web site [51].

### Reconstruction of repetitive sequences

Reconstruction of genomic repeats as well as other computer analysis were performed on an IBM xSeries 226 dual processor server with 4 GB RAM running under the Gentoo Linux operating system. In addition to the programs listed below, several scripts written in Perl were used for data processing and analysis. These scripts are available upon request from authors. The repeat reconstruction was done using TIGR Gene Indices clustering tools (TGICL [25]) employing the following parameters, which were optimized for our dataset by evaluating number and size of resulting clusters, the length of the assembled contigs and the presence of chimeric (misassembled) contig sequences. All-versus-all pairwise similarity scores of the reads were produced by mgblast and parsed using tclust, performing transitive-closure clustering of the read pairs sharing a minimum of 90% similarity over at least 70% of the shorter sequence. The reads within individual clusters were assembled into contigs using tgicl run with the -O '-p 80 -o 40' parameters, specifying overlap percent identity and length cutoff for cap3 assembler. Although most clusters included reads derived from only a single family of genomic repeats, the largest cluster (CL1) was composed from a large number of reads corresponding to several unrelated families of mobile elements. This problem was probably caused by the presence of "hybrid" reads including sequences of two different elements (spanning their insertion sites or recombination breakpoints, see also Discussion). To separate these unrelated sequences, the contigs assembled from CL1 were further grouped into sub-clusters using sclust (parameters HEAVY = 3010 SCORE = 150), which performs weighted seeded clustering based on mutual similarities of the contigs. The programs mgblast, tclust, tgicl, cap3 and sclust are included in the TGICL package and the detailed description of parameters can be obtained from the program help.

The assembled contigs were labeled based on the cluster they originated from (CL [number]Contig [number]); in case of CL1 the name also included sub-cluster number (SCL [number]_CL1Contig [number]). The contig sequences were characterized by calculating their average read depth (RD) and genome representation (GR = RD × contig length). The genome representation was also calculated for individual clusters by summing GR values of the corresponding contigs. Contigs from the clusters representing at least 0.01% of the pea genome (GR > 3,300) were subjected to sequence similarity searches (blastn, blastx) against GenBank database and to detection of conserved protein domains using RPS-BLAST [52] in order to identify the type and family of the repeat they originated from. Satellite repeats were identified by detection of tandem subrepeats within the contig sequences using dotter [53]. Total proportion of identified repeats in the pea genome was calculated by summing the GR of the clusters assigned to the same type of repeat. Detailed information about cluster characterization is available from Additional file 3. All contig sequences with their RD and GR values can be downloaded from our website; alternatively, the contigs can be selected by sequence similarity searches against user-provided queries [51].

### Sequence analysis

Phylogenetic analysis of retroelements was done using multiple sequence alignment of reverse transcriptase domains and a phylogenetic tree was calculated by neighbor-joining method (bootstrap values calculated from 1000 replicates) using the ClustalX program [54]. Reverse transcriptase sequences used for the analysis were extracted mostly from plant retrotransposon sequences downloaded from RepBase [55,56]. Graphical representation of the tree was drawn and edited using ATV Forester [57], TreeEdit [58] and Jalview programs [59]. Copy number (CN) estimation based on RT domains was calculated using formula CN = RD_rt _× 129 × L1/L2, where RD_rt _is the average read depth within the reverse transcriptase domain, L1 is the length of the reverse transcriptase domain in particular contig and L2 is the length of the full size domain. Contigs bearing only partial reverse transcriptase domains and therefore missing in the phylogenetic tree were assigned to appropriate retrotransposon group based on their best Blast hits to elements shown in the tree (Additional files 4 &5).

An alternative approach for copy number estimation of Ogre elements was based on analyzing contigs including the Ogre-specific PBS sequence complementary to tRNA_Arg _[26]. Contig assemblies (ACE files produced by cap3 assembler) were visualized using clview [25] and average read depth over the PBS site was calculated for each contig. The contig sequences were compared using dotter and grouped into three subfamilies according to similarity of the sequences surrounding the PBS. The number of PBS-containing reads was summed for each subfamily and used to calculate its genomic copy number.

Microsatellite and telomeric repeats were identified directly in a set of individual 454 reads instead of in the contigs. The reads separated with runs of "N" (to avoid fusing adjacent sequences) were concatenated into a single sequence and analyzed using Tandem Repeats Finder [60]. The results were sorted and evaluated using TRAP [61].

Comparative analysis of the repeat composition in pea, soybean (*Glycine max*) and barrel medic (*Medicago truncatula*) was performed using our set of 319,402 pea 454 reads, a set of recently published 718,589 soybean 454 reads [19], and 197,570 *M. truncatula *BAC-end sequences. The soybean and *M. truncatula *sequences were downloaded from the NCBI Trace Archive [48] and from TIGR [62], respectively. Sequence similarities were identified by blastn searches between 454 reads from pea and soybean, and between either pea or soybean 454 reads and *M. truncatula *BAC-end sequences. The 454 reads producing blastn hits with E-value of 1e^-10 ^or lower to sequences from the other species were considered to include sequences shared between these genomes. For each of these reads, the number of hits to other genome sequences and average blastn bit score of these hits were determined and plotted using Mgraph (created by Louis Gonzalez and Christine Deroo, available from [63]). Grouping the reads according to the repeat type was done by similarity searches to known sequences performed as described above.

### Fluorescence in situ hybridization (FISH)

Hybridization probes for potential satellite repeats were prepared by PCR amplification from pea genomic DNA using primer pairs designed according to the contig sequences including tandem subrepeats (Table 1 and Additional file 6). The PCR was performed in 30 μl of reaction mix (1× PCR buffer, 1.5 mM MgCl_2_, 0.2 mM dNTPs, 0.2 μM primers, 5 U of Taq polymerase (Promega), 0.2–20 ng of template DNA) for 25 cycles of 1 min at 94°C, 1 min at 50°C and 3 min at 72°C, preceded by initial denaturation (3 min at 94°C) and followed by final extension step (10 min at 72°C). Reaction products were resolved on agarose gel electrophoresis and bands correspoding to the repeat monomers or short multimers (< 800 bp) were excised from ladders of the amplified fragments. The excised DNA was purified and used as a template in PCR labeling by biotin-dUTP as described [20]. 5' biotin-labeled oligonucleotide probes for alternative telomeric repeats (CCTAA)_8_, (CCTAAA)_7 _and controls (CGTAA)_8_, (CGTAAA)_7_-CG were purchased from Bioneer Europe. FISH experiments were carried out on chromosome squash preparations [64] obtained from root tip meristems synchronized as described [65]. The chromosome preparations were aged for 5–60 days and post-fixed in 4% formaldehyde/2 × SSC for 10 min at 25°C. The hybridization was performed as described [64] with the following modifications. Probe concentration in the hybridization mix was 0.2 μM for oligonucleotide and 0.5 ng/μl for PCR-amplified fragments. Formamide concentrations in hybridization and washing solutions and incubation temperatures were adjusted according to the probe sequences to achieve 90% stringency for the oligonucleotide probes (Tm calculated using Exiqon Tm prediction 1.1 software [66]) and 80% stringency for the satellite repeat probes. Stringency for both types of probes was calculated according to [64]. Biotin-labeled probes were detected with streptavidin-Alexa Fluor 568 (Invitrogen) and individual chromosome types were distinguished by co-hybridization with Alexa Fluor 488-labeled PisTR-B probe, producing characteristic patterns on each pea chromosome [20]. Chromosomes were counterstained with 4,6-diamino-2-phenylindole (DAPI) and observed using a Nikon Eclipse-600 epifluorescence microscope equipped with a CCD camera. The signals were collected using appropriate filter sets and LUCIA software (Laboratory Imaging).

## Authors' contributions

JM conceived the study and carried out most of the bioinformatics analysis. PN participated in repeat identification and conducted phylogenetic analysis of retroelements. AN carried out FISH experiments. All authors contributed to the manuscript preparation and approved its final version.

## Supplementary Material

Additional file 1 **Repetitive sequences and their copy number estimates used for the evaluation of repeat representation in 454 data**. The table provides GenBank accession numbers, descriptions and experimentally determined copy numbers of selected pea repeats together with calculations of their genomic abundance based on the 454 data. Click here for file

Additional file 2 Sorted list of 50 reconstructed contigs with the highest genome representation. Click here for file

Additional file 3 Assignment of clusters of reconstructed contigs to individual repeat families. Click here for file

Additional file 4 **Phylogenetic analysis of Ty3/gypsy elements based on their reverse transcriptase domains**. Phylogenetic trees show relationship of reverse transcriptase domains identified in pea 454 contigs to those from selected plant retroelements. Only contigs bearing complete or marginally truncated (marked with asterisks) RT domains were used for analysis and their names and positions are labeled with yellow boxes. Contigs containing only partial RT sequences were assigned to the trees based on their sequence similarity to full-length domains (labeled as "best hit"). Copy numbers of elements belonging to individual clades are provided in Figure 4. Click here for file

Additional file 5 **Phylogenetic analysis of Ty1/copia elements based on their reverse transcriptase domains**. Phylogenetic trees show relationship of reverse transcriptase domains identified in pea 454 contigs to those from selected plant retroelements. Only contigs bearing complete or marginally truncated (marked with asterisks) RT domains were used for analysis and their names and positions are labeled with yellow boxes. Contigs containing only partial RT sequences were assigned to the trees based on their sequence similarity to full-length domains (labeled as "best hit"). Copy numbers of elements belonging to individual clades are provided in Figure 4. Click here for file

Additional file 6 PCR primers used for preparation of FISH probes for reconstructed tandem repeats. Click here for file
