# Supplementary material for: Repetitive DNA in the pea (Pisum sativum L.) genome: comprehensive characterization using 454 sequencing and comparison to soybean and Medicago truncatula
Source: BMC Genomics. 2007 Nov 21;8:427. doi: 10.1186/1471-2164-8-427 (PMC2206039; doi:10.1186/1471-2164-8-427)
Supplement: Additional file 1 — Repetitive sequences and their copy number estimates used for the evaluation of repeat representation in 454 data. The table provides GenBank accession numbers, descriptions and experimentally determined copy numbers of selected pea repeats together with calculations of their genomic abundance based on the 454 data. [file 1471-2164-8-427-S1.pdf]

Additional file 1 – Pea sequences used for evaluation of repeat representation in 454 data

Sequence [(a)]	Repeat type	GenBank accession	Length [bp]	Experiment [(a)] copies/1C	454 data [(b)] hit depth	454 data [(b)] copies/1C	Exp. / 454 [(c)]
Psat1_1	gag-pol	AF155741	468	33000	138	17802	1.85
Psat1_5	gag-pol	AF155745	543	22000	172	22188	0.99
Psat1_9	gag-pol	AF155749	659	15000	166	21414	0.70
Psat2_2	gag-pol	AF155742	495	26000	152	19608	1.33
Psat2_13		AF155752	502	8000	65	8385	0.95
Psat2_14	gag-pol	AF155753	585	17000	91	11739	1.45
Psat2_33	gag-pol (Ogre)	AF155770	512	29000	162	20898	1.39
Psat3_3	LTR (Ogre)	AF155743	351	26000	114	14706	1.77
Psat3_10	LTR (Ogre)	AF318289	469	29000	124	15996	1.81
Psat3_16	LTR (Ogre)	AF155754	359	13000	54	6966	1.87
Psat3_18	LTR (Ogre)	AF155756	375	30000	94	12126	2.47
Psat3_26	LTR (Ogre)	AF155763	517	18000	115	14835	1.21
Psat4		AF155744	957	16000	137	17673	0.91
Psat6	gag-pol	AF155746	607	19000	74	9546	1.99
Psat7	gag-pol	AF155747	469	39000	143	18447	2.11
Psat8		AF155748	663	20000	110	14190	1.41
Psat11		AF155750	899	8000	42	5418	1.48
Psat12	gag-pol	AF155751	405	7000	83	10707	0.65
Psat17		AF155755	542	5000	26	3354	1.49
Psat19	gag-pol	AF155757	168	15000	140	18060	0.83
Psat20		AF155758	325	6000	38	4902	1.22
Psat22	LTR	AF155759	937	2000	21	2709	0.74
Psat23	LTR	AF155760	212	16000	44	5676	2.82
Psat24	LTR (copia)	AF155761	1134	13000	157	20253	0.64
Psat25		AF155762	1023	5000	41	5289	0.95
Psat27_27	LTR (Ogre)	AF155764	1168	8000	94	12126	0.66
Psat27_29	LTR (Ogre)	AF155766	2089	10000	65	8385	1.19
Psat28	gag-pol	AF155765	195	18000	224	28896	0.62
Psat30_30		AF155767	2961	9000	133	17157	0.52
Psat30_31		AF155768	2591	10000	132	17028	0.59
Psat32	gag-pol (Peabody)	AF155769	490	10000	92	11868	0.84
Psat34	gag-pol	AF155771	149	1000	15	1935	0.52
PIGY-1	gag-pol	AY299398	4500	2500	23	2967	0.84
PisTR-A	Tandem repeat	consensus [(a)]	211	20000	135	17415	1.15
Stow-Ps	MITE	consensus [(a)]	279	1000(-10000)	8	1032	0.97
Zaba-Ps2	MITE	AY204902	189	50 (-500)	0 (1 hit)		

[a] For further information about the repeat sequences and their experimental copy number estimates see Neumann et al. (2001) *Genome* 44: 716-728 (all Psat repeats and PisTR-A), Macas et al. (2003) *Mol. Genet. Genomics* 269: 624-631 (Zaba MITEs), Neumann et al. (2005) *Mol. Genet. Genomics* 273: 43-53 (PIGY) and Macas et al. (2005) *Genome* 48: 831-839 (Stowaway MITEs).

[b] Repeat repesentation in 454 sequence reads was estimated by runnig blastn similarity searches against a database of 454 reads and determining average number of highly significant hits (E-value <= 10^{-10}) along the query sequence (“hit depth“). Estimates of the repeat copy numbers per haploid genome (1C) were obtained by multiplying hit depth values by 129 (the total length of all 454 reads corresponded to 1/129 of pea haploid genome size).

[c] Ratio of copy number estimates from experimental and 454 data.
