# Supplementary figures and images for: Repetitive DNA in the pea (Pisum sativum L.) genome: comprehensive characterization using 454 sequencing and comparison to soybean and Medicago truncatula
Source: BMC Genomics. 2007 Nov 21;8:427. doi: 10.1186/1471-2164-8-427 (PMC2206039; doi:10.1186/1471-2164-8-427)

Ty3/gypsy-like retrotransposons

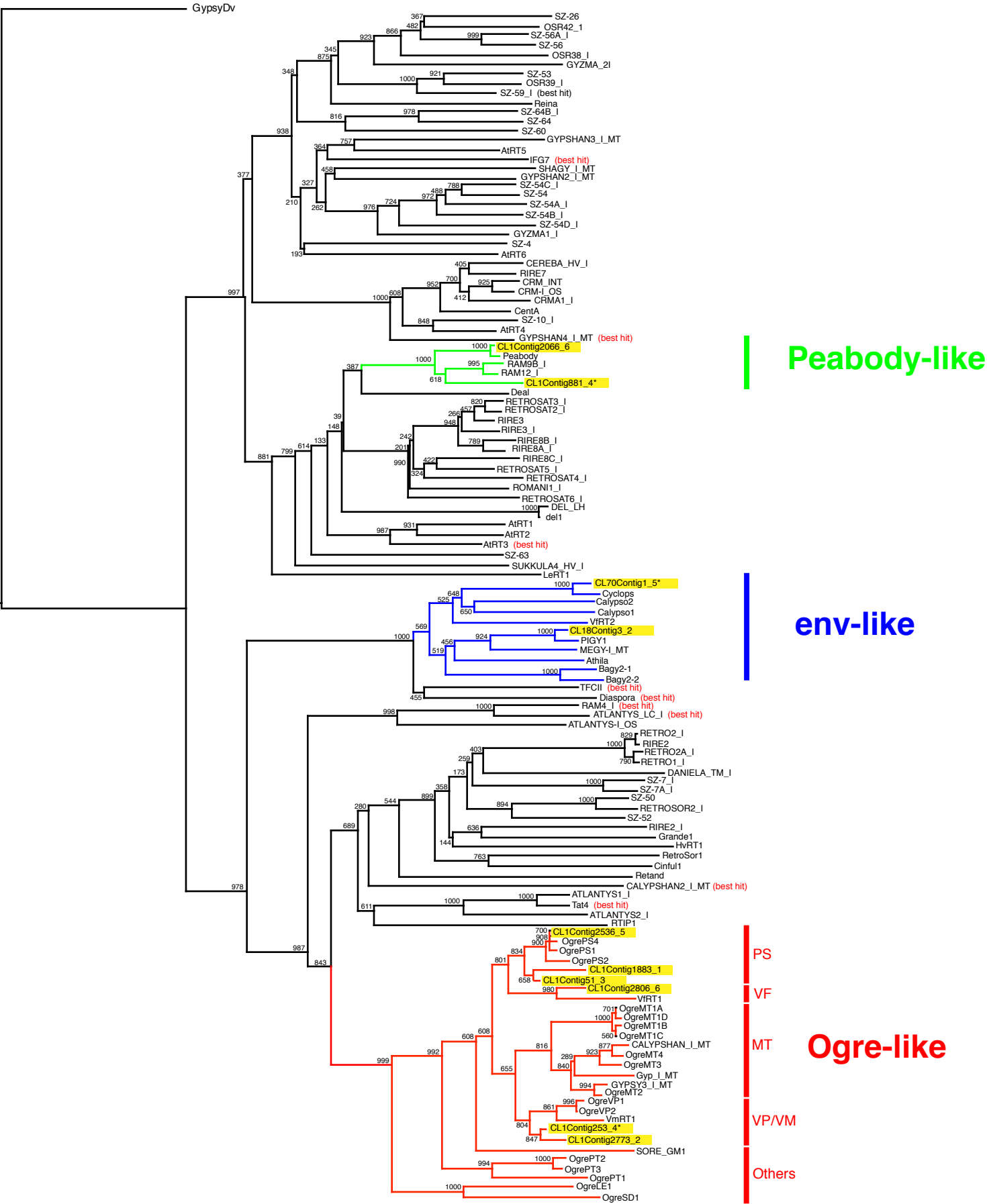

Supplement: Additional file 4 — Phylogenetic analysis of Ty3/gypsy elements based on their reverse transcriptase domains. Phylogenetic trees show relationship of reverse transcriptase domains identified in pea 454 contigs to those from selected plant retroelements. Only contigs bearing complete or marginally truncated (marked with asterisks) RT domains were used for analysis and their names and positions are labeled with yellow boxes. Contigs containing only partial RT sequences were assigned to the trees based on their sequence similarity to full-length domains (labeled as "best hit"). Copy numbers of elements belonging to individual clades are provided in Figure 4. [file 1471-2164-8-427-S4.pdf]

Ty1/copia-like retrotransposons

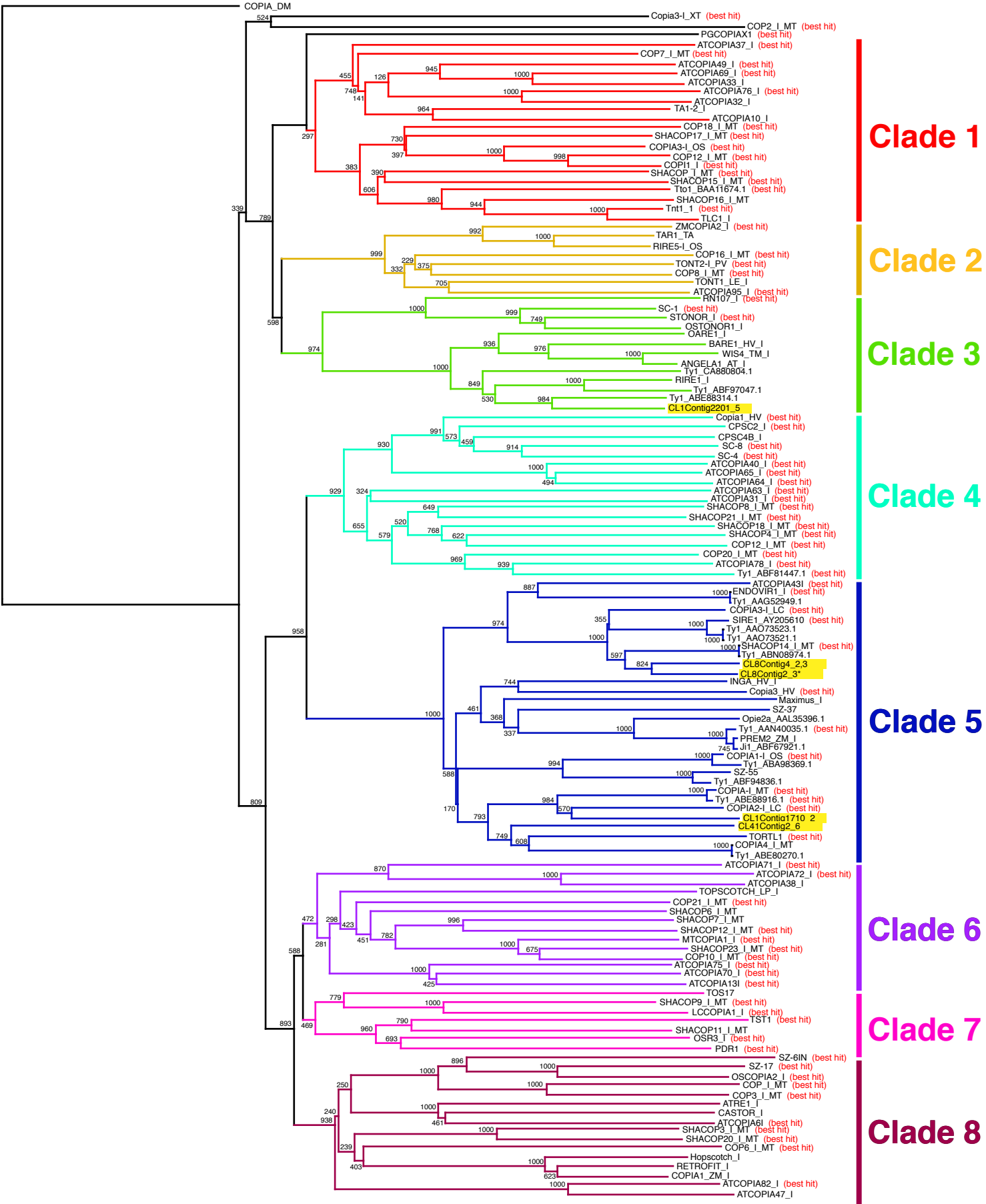

Supplement: Additional file 5 — Phylogenetic analysis of Ty1/copia elements based on their reverse transcriptase domains. Phylogenetic trees show relationship of reverse transcriptase domains identified in pea 454 contigs to those from selected plant retroelements. Only contigs bearing complete or marginally truncated (marked with asterisks) RT domains were used for analysis and their names and positions are labeled with yellow boxes. Contigs containing only partial RT sequences were assigned to the trees based on their sequence similarity to full-length domains (labeled as "best hit"). Copy numbers of elements belonging to individual clades are provided in Figure 4. [file 1471-2164-8-427-S5.pdf]
