# Supplementary material for: Repetitive DNA in the pea (Pisum sativum L.) genome: comprehensive characterization using 454 sequencing and comparison to soybean and Medicago truncatula
Source: BMC Genomics. 2007 Nov 21;8:427. doi: 10.1186/1471-2164-8-427 (PMC2206039; doi:10.1186/1471-2164-8-427)
Supplement: Additional file 6 — PCR primers used for preparation of FISH probes for reconstructed tandem repeats. [file 1471-2164-8-427-S6.pdf]

PCR primers used for preparation of FISH probes for reconstructed tandem repeats

Repeat	Primers (5'->3')
TR-1	TTCAACAACGGCAAAACATC,<br>CTATATACTTTTAAGATCAATGG
TR-2	GAAGGCCATCCAAGTTTCAT,<br>ATGACCTATAATCTTTCACC
TR-3	ATCTCATTTATATAATGAGG,<br>AATATACATATKACAACCTC
TR-4	GTCTTAGATTCAATTAAGGT,<br>AAAATTGAATGAGACTCTTC
TR-5	CGAGTGTTGAAAATCAATAC,<br>TCATTTCTTTGTCAATACTC
TR-6	TGATTTCAATGAATAACTCG,<br>ATATGATGTATGGGTTATTC
TR-7	GTATTTGTATACATGTTTTACC,<br>AGGATAAATGTGGTACCTAC
TR-8	TTGCCAGCGGAATTATCAC,<br>AATAACACCAAACACCACTC
TR-9	ATACCATAATAYAATGTGAATAT,<br>CAAATTTTAGGTTTCTTCATC
TR-10	TTATTATCAGAACCATGAAC,<br>TGTAAAAGACTTCTTTATGG
TR-11	TRTTACTTAGCTACTTAATG,<br>CCATCCTATAATGTTCAAAG
TR-12	AATAGCCTTTCCTTAGTGTC,<br>GATTTAATCGACATTGGAAG
TR-14	CAACCCAAAACAAAGAAATTAC,<br>GGTTTTGGCTCTTCGTAATC
TR-17	AGTCAATTACGGTGTAGGTG,<br>TACATTGATTTGTGTGCAAG
